# TRPV_1_ in Brain Is Involved in Acetaminophen-Induced Antinociception

**DOI:** 10.1371/journal.pone.0012748

**Published:** 2010-09-17

**Authors:** Christophe Mallet, David A. Barrière, Anna Ermund, Bo A. G. Jönsson, Alain Eschalier, Peter M. Zygmunt, Edward D. Högestätt

**Affiliations:** 1 Clermont Université, Université d'Auvergne, Pharmacologie fondamentale et clinique de la douleur, Clermont-Ferrand, France; 2 Inserm, U 766, Clermont-Ferrand, France; 3 Department of Occupational and Environmental Medicine, Lund University, Lund, Sweden; 4 Department of Clinical Chemistry and Pharmacology, Lund University and Lund University Pain Research Centre, Lund, Sweden; Tokyo Institute of Psychiatry, Japan

## Abstract

**Background:**

Acetaminophen, the major active metabolite of acetanilide in man, has become one of the most popular over-the-counter analgesic and antipyretic agents, consumed by millions of people daily. However, its mechanism of action is still a matter of debate. We have previously shown that acetaminophen is further metabolized to N-(4-hydroxyphenyl)-5Z,8Z,11Z,14Z -eicosatetraenamide (AM404) by fatty acid amide hydrolase (FAAH) in the rat and mouse brain and that this metabolite is a potent activator of transient receptor potential vanilloid 1 (TRPV_1_) *in vitro*. Pharmacological activation of TRPV_1_ in the midbrain periaqueductal gray elicits antinociception in rats. It is therefore possible that activation of TRPV_1_ in the brain contributes to the analgesic effect of acetaminophen.

**Methodology/Principal Findings:**

Here we show that the antinociceptive effect of acetaminophen at an oral dose lacking hypolocomotor activity is absent in FAAH and TRPV_1_ knockout mice in the formalin, tail immersion and von Frey tests. This dose of acetaminophen did not affect the global brain contents of prostaglandin E_2_ (PGE_2_) and endocannabinoids. Intracerebroventricular injection of AM404 produced a TRPV_1_-mediated antinociceptive effect in the mouse formalin test. Pharmacological inhibition of TRPV_1_ in the brain by intracerebroventricular capsazepine injection abolished the antinociceptive effect of oral acetaminophen in the same test.

**Conclusions:**

This study shows that TRPV_1_ in brain is involved in the antinociceptive action of acetaminophen and provides a strategy for developing central nervous system active oral analgesics based on the coexpression of FAAH and TRPV_1_ in the brain.

## Introduction

In 1948, Brodie and Axelrod demonstrated that acetaminophen is the major active metabolite of acetanilide in man [1]. Since then, acetaminophen has become one of the most popular over-the-counter analgesic and antipyretic agents, consumed by millions of people daily. Although acetaminophen shares some pharmacological properties with the cyclooxygenase (COX) inhibitors, its anti-inflammatory effect is weak and it is devoid of the typical adverse effects of the COX inhibitors, indicating partly different mechanisms of action. The additive analgesic effects of acetaminophen and COX inhibitors, as observed in several clinical trials [2], may also reflect non-overlapping mechanisms of action. Furthermore, acetaminophen is active in human and animal models of acute non-inflammatory pain, which are considered insensitive to COX inhibitors [3]–[11].

Acetaminophen is a high dose analgesic and antipyretic agent, undergoing extensive metabolism in the body. We have recently shown that acetaminophen is metabolized by FAAH to the N-acylphenolamine AM404 in the rodent nervous system and that inhibitors of FAAH prevent the antinociceptive effect of acetaminophen in rat [6], [12]. This compound, which possesses analgesic activity in models of nociceptive and neuropathic pain [13]–[20], was originally proposed to be an inhibitor of cellular uptake and degradation of anandamide [21]. However, subsequent studies have shown that AM404 is also a potent activator of the capsaicin receptor TRPV_1_ and an inhibitor of COX *in vitro* [12], [22], [23]. Interestingly, some of its antinociceptive effects were sensitive to the TRPV_1_ blocker capsazepine [13], [14]. Thus, the downstream target(s) for the antinociceptive effect of acetaminophen is an open question.

This study explores the role of TRPV_1_ in the antinociceptive effect of acetaminophen in rodent models of chemical (formalin test), thermal (tail immersion test) and mechanical (von Frey test) pain. These nociceptive tests are intact in TRPV_1_^−/−^ mice and sensitive to acetaminophen, but considered to be partially or completely insensitive to COX inhibitors [3], [6], [8], [9], [24], [25]. The levels of prostanoids and endocannabinoids in brain were also measured to address the possibility that acetaminophen or its metabolites interferes with COX or the elimination of endocannabinoids in this organ. We found that acetaminophen at a dose that did not affect spontaneous locomotor activity or the contents of PGE_2_ and endocannabinoids in brain produced a robust antinociceptive effect that was absent in TRPV_1_^−/−^ mice and abolished by systemic or intracerebroventricular injection of capsazepine.

## Results

**Figure 1:**
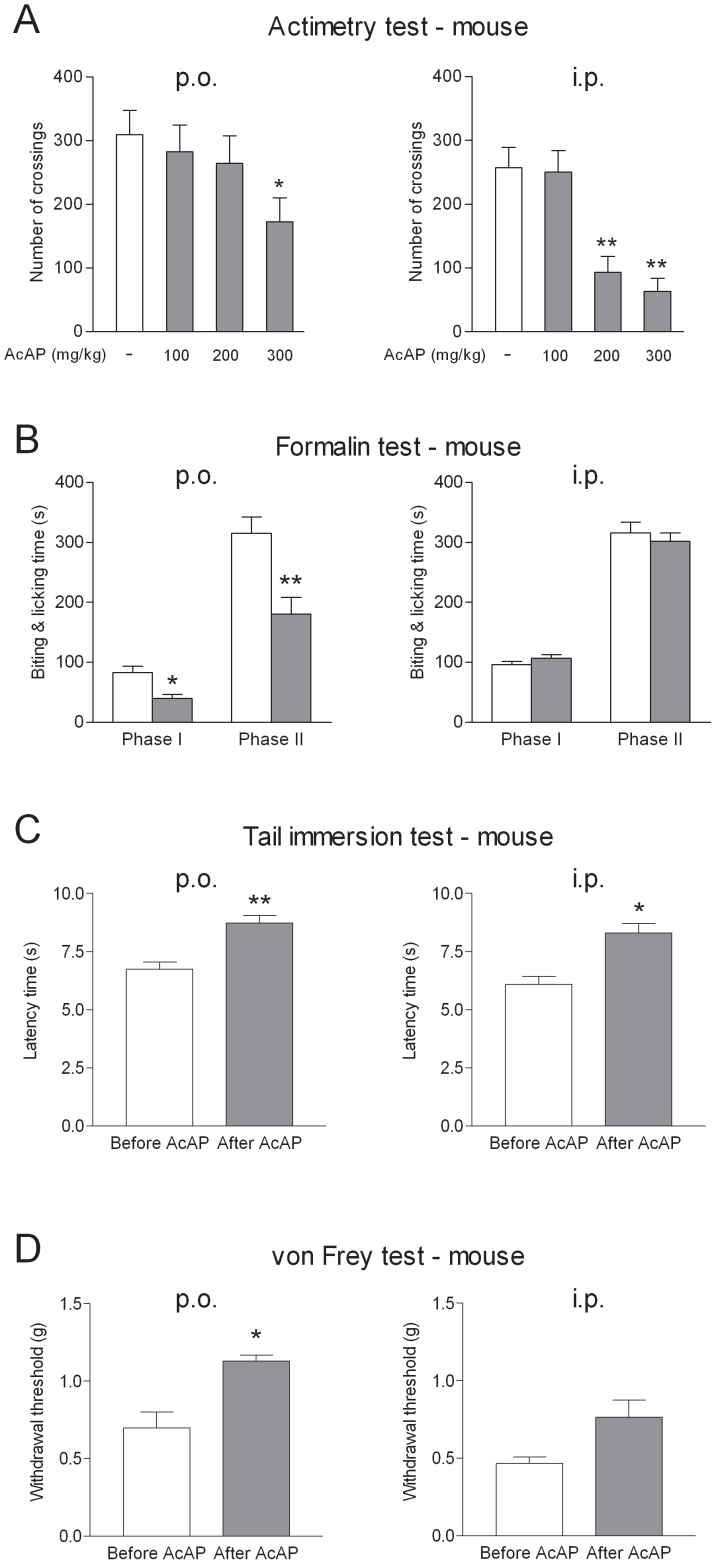
Dose-effect relationship of acetaminophen on locomotor activity and nociception in mice. Left and right panels of the figure show the effects of acetaminophen after oral (p.o.) and intraperitoneal (i.p.) administration, respectively. (A) Animals were placed in actimetry boxes (Actisystem, Apelex) and their movements were assessed by determining the number of crossings of light beams during 15 min. Intraperitoneal doses induced a higher reduction in spontaneous activity than oral doses. The test was performed 20 min after acetaminophen administration. Data are given as mean ± SEM (n = 6). *P<0.05, **P<0.01 compared to vehicle. (B–D) Mice were submitted to chemical (B), thermal (heat; C) and mechanical (D) stimuli before and 20 min after oral or intraperitoneal administration of acetaminophen at the highest dose that was without effect on locomotor activity (200 mg/kg p.o. and 100 mg/kg i.p.). Data are given as mean ± SEM (n = 6). *P<0.05, **P<0.01 compared to vehicle (A, B) or before treatment (C, D).

An initial set of experiments was designed to establish a dose and a route of administration (oral versus parenteral) of acetaminophen that produced robust responses in nociceptive tests without impairing motor activity. These experiments showed that an oral dose of 200 mg/kg in mice produced robust antinociceptive effects in the formalin, the tail immersion and the von Frey tests, but did not affect the spontaneous locomotor activity (Fig. 1, left panel). Such a clear separation of antinociceptive and hypolocomotor effects was not observed when acetaminophen was administered intraperitoneally (Fig. 1, right panel). Based on these studies, we selected an oral dose of 200 mg/kg in the subsequent nociceptive tests in mice. As shown previously, oral doses up to 300 mg/kg do not reduce the spontaneous locomotor activity in the rat [6].

**Figure 2:**
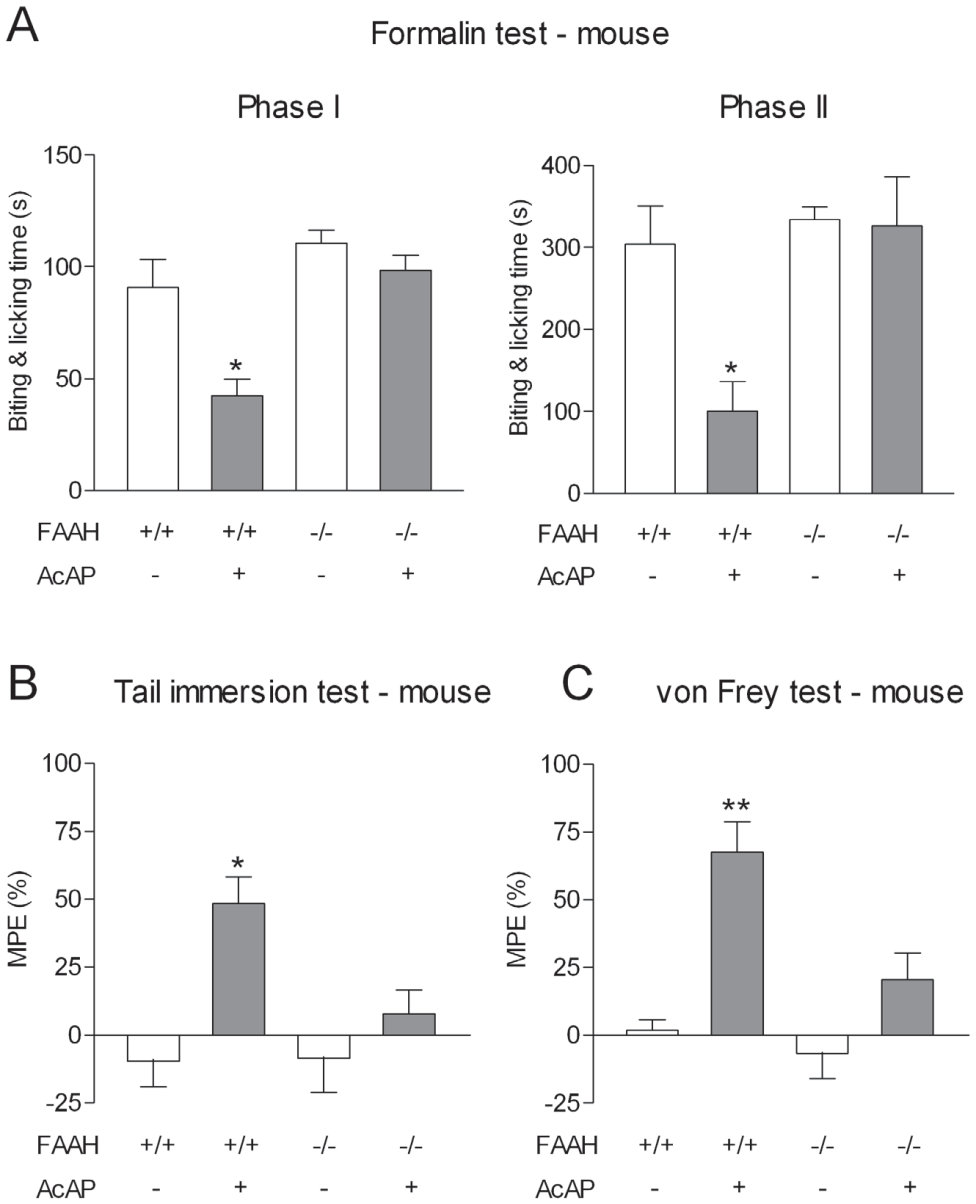
The antinociceptive effect of acetaminophen is dependent on FAAH. The effect of acetaminophen (AcAP; 200 mg/kg p.o.) was assessed in (A) the formalin test (B) the tail immersion test and (C) the von Frey test. In FAAH^−/−^ mice, acetaminophen failed to produce antinociception, while it was effective in FAAH^+/+^ mice in the same tests. All tests were performed 20 min after acetaminophen administration. In the tail immersion and von Frey test tests, results are expressed as MPE (%): [(post-treatment score – pre-treatment score)/(cut-off value – pre-treatment score) ×100]. Basal pre-treatment threshold responses were 7.73±0.32 and 8.05±0.29 s in the tail immersion test and 0.55±0.03 and 0.62±0.06 g in the von Frey test for FAAH^+/+^ and FAAH^−/−^ mice, respectively. Data are presented as mean ± SEM (n = 6–8 per group). *P<0.05, **P<0.01 compared to vehicle.

Fatty acid amide hydrolase is a key enzyme in the metabolism of acetaminophen to potentially analgesic N-acylphenolamines, including AM404 [12]. We therefore examined the antinociceptive effect of acetaminophen in FAAH^−/−^ mice and wild-type littermates in the various tests. In FAAH^+/+^ mice, acetaminophen reduced the time spent biting and licking during the first and the second phases of the formalin test and increased the withdrawal thresholds in the tail immersion and the von Frey tests (Fig. 2 A, B and C). However, acetaminophen failed to produce antinociception in the same tests in FAAH^−/−^ mice (Fig. 2 A, B and C). The endocannabinoids anandamide and 2-arachidonoylglycerol are effectively metabolized by FAAH and monoacylglycerol lipase, respectively, and thus we considered the possibility that acetaminophen increases the brain levels of analgesic endocannabinoids by inhibition of their metabolism. However, neither oral nor intraperitoneal administration of acetaminophen affected the global content of the endocannabinoids in the mouse or rat brain (Table S1, Fig. S1). Collectively, our results show that the antinociceptive action of acetaminophen is dependent on FAAH, but not mediated by changes in the levels of endocannabinoids in brain.

**Figure 3:**
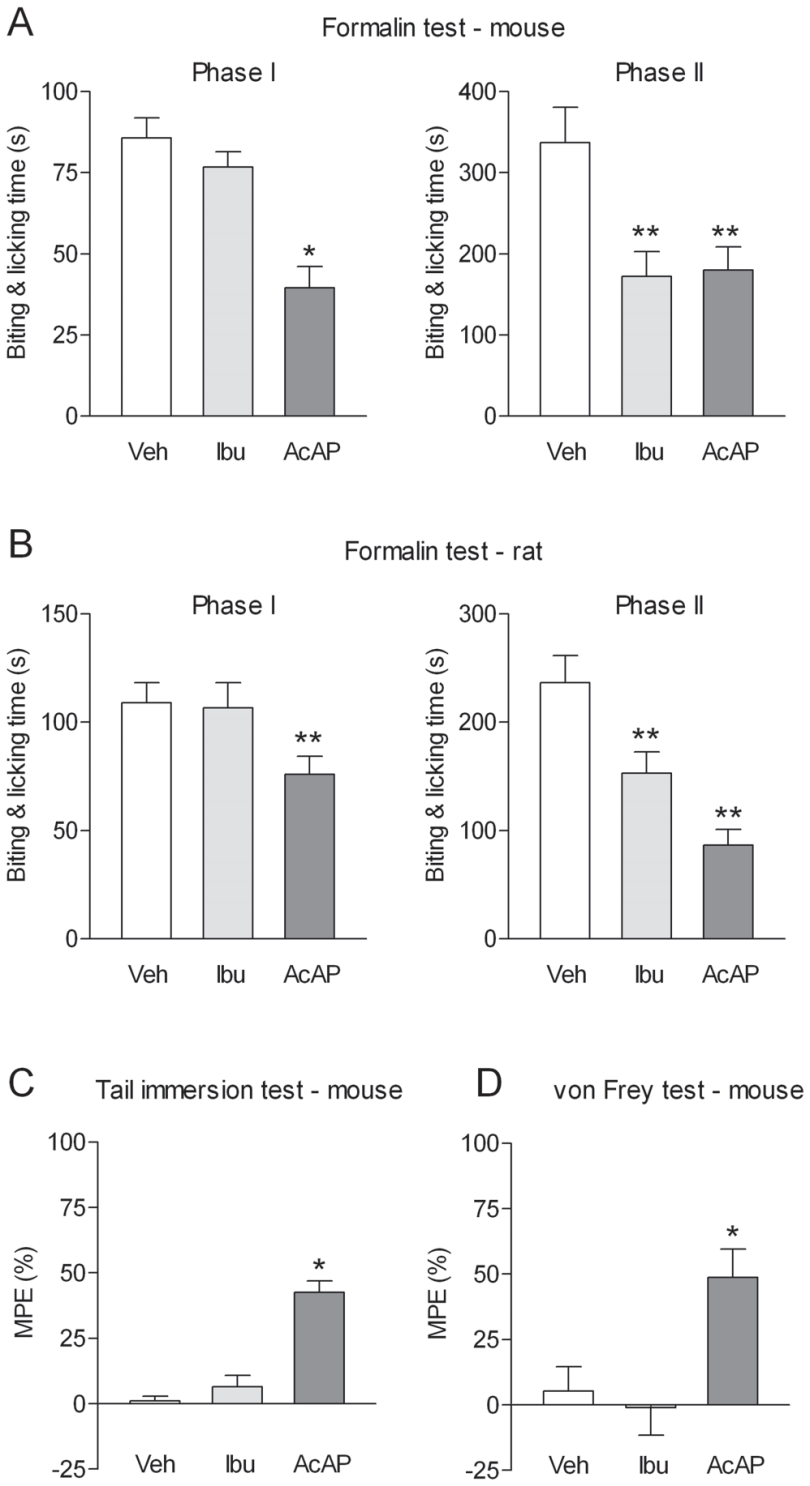
Comparison of the effects of acetaminophen and ibuprofen in various pain tests. (A) The antinociceptive effects of oral administration of acetaminophen (AcAP; 200 mg/kg in mice and 300 mg/kg in rats) and intraperitoneal injection of ibuprofen (Ibu; 100 mg/kg) were assessed in (A,B) the formalin test, (C) the tail immersion test and (D) the von Frey test. In the first phase of the formalin test as well as the von Frey and the tail immersion tests, acetaminophen produced an antinociceptive effect, whereas ibuprofen was ineffective. Acetaminophen and ibuprofen inhibited the nociceptive behavior during the second phase of the formalin test. In the tail immersion and von Frey test tests, results are expressed as MPE (5): [(post-treatment score – pre-treatment score)/(cut-off value – pre-treatment score) ×100]. Basal pre-treatment threshold responses were 7.29±0.43 s in the tail immersion test and 0.67±0.08 g in the von Frey test, respectively. All tests were performed 20 min after acetaminophen or ibuprofen administration. Data are presented as mean ± SEM (n = 6–8 per group). *P<0.05, **P<0.01 compared to vehicle.

Substantial levels of AM404 were detected in brain after systemic administration of acetaminophen and *p*-aminophenol in rodents [12]. This together with our previous finding that AM404 inhibits COX *in vitro* [12] prompted us to measure the content of PGE_2_ in mouse brain after oral administration of acetaminophen at a dose of 200 mg/kg. However, no difference in the PGE_2_ contents was observed between acetaminophen-treated (3.0±1.1 nmol/mg protein) and vehicle-treated (3.4±1.3 nmol/mg protein) animals (n = 9). We next compared the effects of acetaminophen and the competitive COX inhibitor ibuprofen in the various nociceptive tests in mice and rat (Fig. 3). Ibuprofen was administered intraperitoneally at a dose of 100 mg/kg that produced more than 90% inhibition of the PGE_2_ content in the mouse brain (Fig. S2 A). In both mice and rats, acetaminophen and ibuprofen reduced the nocifensive behavior during the second phase of the formalin test (Fig. 3 A and B). However, only acetaminophen inhibited the first phase of the formalin test (Fig. 3 A and B). In the tail immersion and the von Frey tests in mice, acetaminophen, but not ibuprofen, increased the withdrawal thresholds (Fig. 3 C and D). The different pharmacological profiles of acetaminophen and ibuprofen in the biochemical and behavioral assays indicate that acetaminophen has antinociceptive effects that cannot be attributed to inhibition of COX.

**Figure 4:**
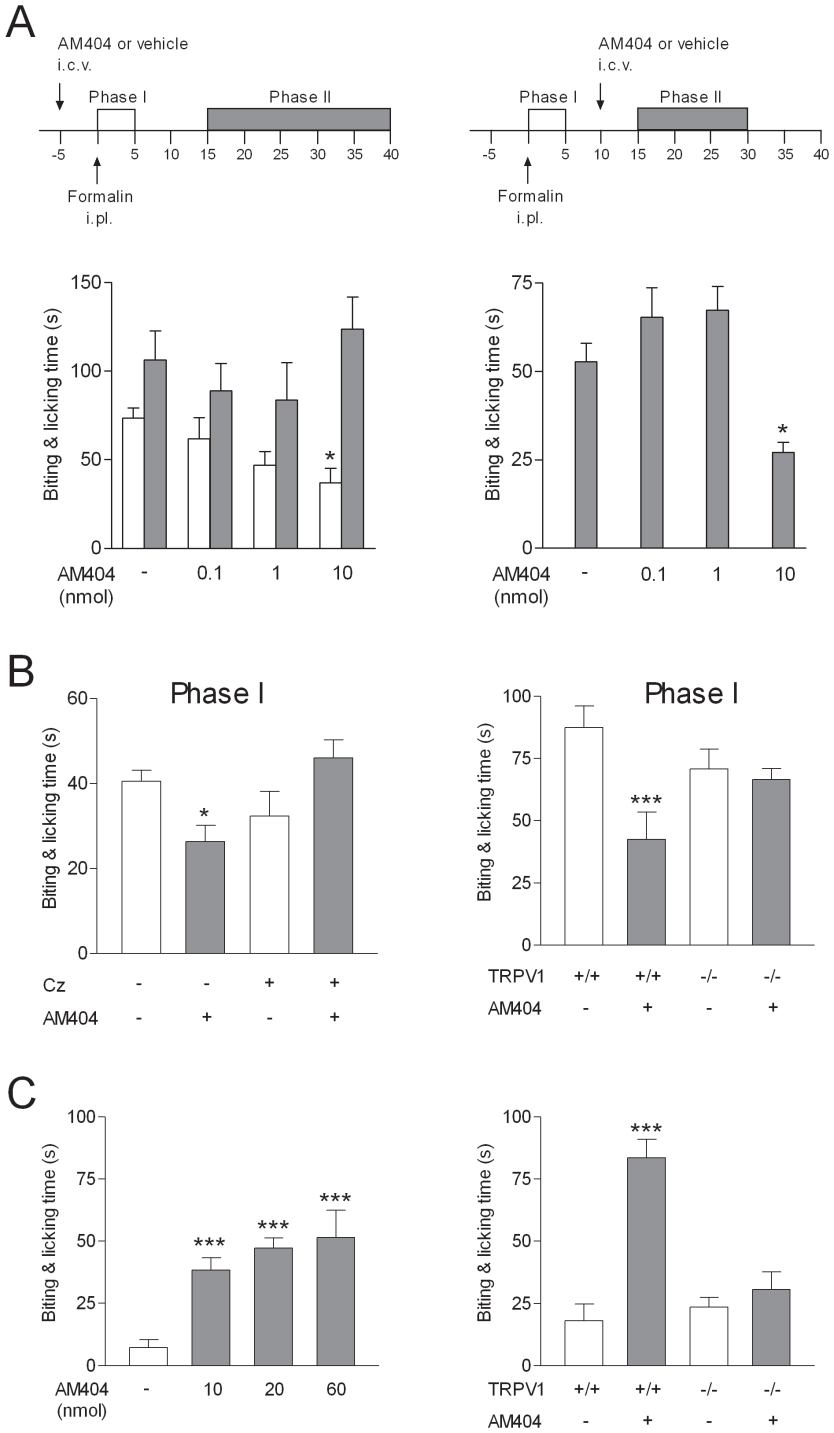
The acetaminophen metabolite AM404 produces antinociception by activation of TRPV_1_ in the brain. (A) Intracerebroventricular (i.c.v.) injection of AM404 (10 nmol) 5 min before injection of formalin into the mouse hind paw decreased biting and licking during the first, but not the second phase of the formalin test (n = 5–6). When AM404 (10 nmol) was administered 10 min after formalin, it also decreased the second phase (15 to 30 min post-formalin) (n = 10–12). (B) The role of TRPV_1_ in the action of AM404 was further investigated on the first phase of the formalin test. The effect of AM404 (10 nmol) was inhibited by coinjection of AM404 with capsazepine (Cz; 100 nmol) and absent in TRPV_1_^−/−^ mice (n = 5–8). (C) Injection of AM404 into the mouse hind paw induced a pronounced nocifencive behavior, as recorded during 5 min after the injections (n = 5–6). The nociceptive response to intraplantar injection AM404 (60 nmol) was absent in TRPV_1_^−/−^ mice (n = 6). Data are presented as mean ± SEM. *P<0.05, ***P<0.001 compared to vehicle in wild-type mice.

To understand the potential role of AM404 in the pharmacological action of acetaminophen, we first examined the effect of intracerebroventricular injection of AM404 at different doses in the mouse formalin test. AM404 at a dose of 10 nmol/mouse inhibited the first phase of the formalin test when injected 5 min prior to formalin (Fig. 4 A). The second phase of the formalin test was not affected by this dose of AM404, possibly due to a rapid elimination of AM404 in the brain in the absence of its precursor *p*-aminophenol [12], [26]. However, a significant effect was observed on this phase when AM404 was injected 10 min after formalin (Fig. 4 A). Additional experiments showed that the effect of AM404 on the first phase of the formalin test was absent when AM404 was co-administered with the TRPV_1_ blocker capsazepine (100 nmol/mouse) as well as in TRPV_1_^−/−^ mice (Fig. 4 B). In contrast, intraplantar injection of AM404 at a dose of 10 nmol/mouse or higher produced an acute nocifensive response. This pronociceptive effect was absent in TRPV_1_^−/−^ mice (Fig. 4 C). Taken together, these experiments show that AM404 via activation of TRPV_1_ produces antinociception when injected into the brain, but pronociception when injected into the paw.

**Figure 5:**
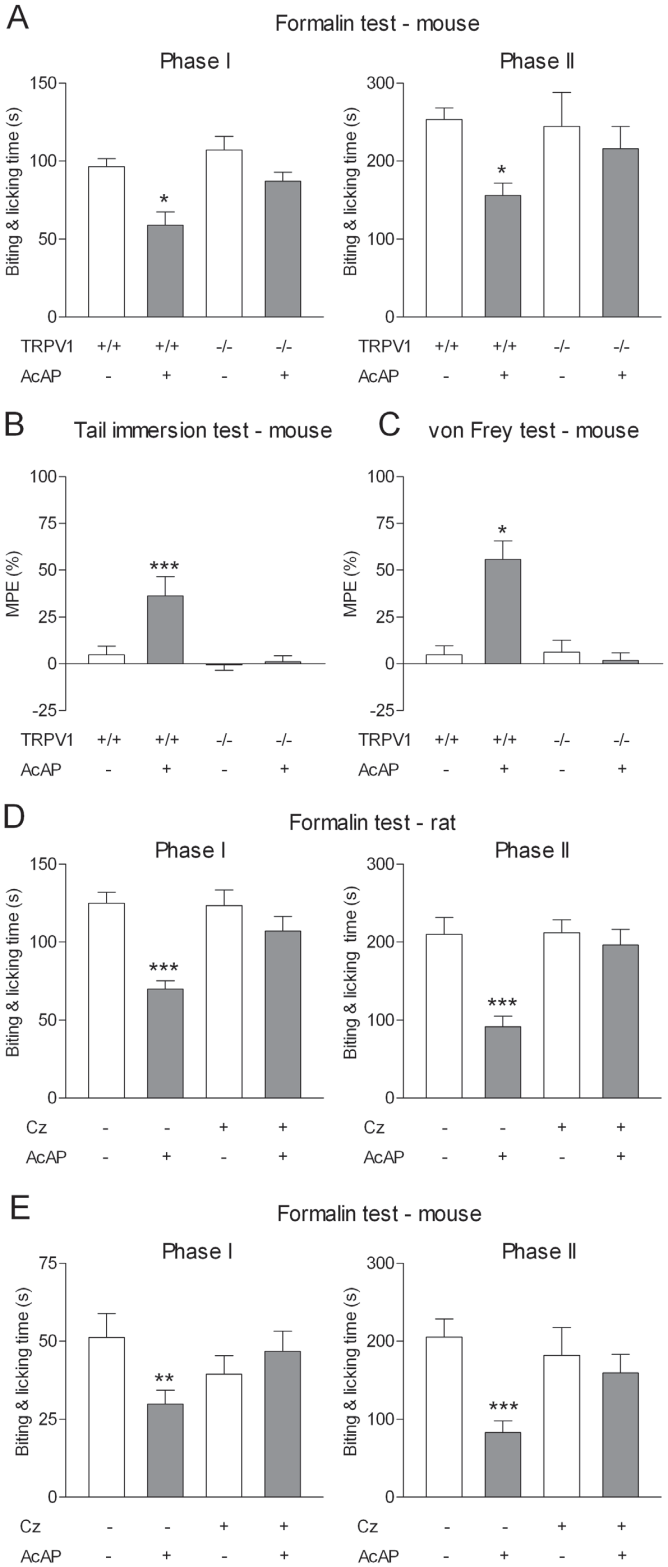
The antinociceptive effect of acetaminophen is dependent on TRPV_1_ in brain. The effect of acetaminophen (AcAP; 200 mg/kg p.o.) was assessed in (A) the formalin test, (B) the tail immersion test and (C) the von Frey test. In TRPV_1_^−/−^ mice, acetaminophen failed to produce antinociception, while it was effective in TRPV_1_^+/+^ mice in the same tests. All tests were performed 20 min after acetaminophen administration. In the tail immersion and von Frey test tests, results are expressed as MPE (%): [(post-treatment score – pre-treatment score)/(cut-off value – pre-treatment score) ×100]. Basal pre-treatment threshold responses were 7.59±0.79 and 8.63±0.88 s in the tail immersion test and 0.67±0.08 and 0.68±0.10 g in the von Frey test for TRPV_1_^+/+^ and TRPV_1_^−/−^ mice, respectively. (D) The effect of acetaminophen (300 mg/kg p.o.) in the formalin test was also examined in rats pre-treated with capsazepine (Cz; 10 mg/kg i.p.) or vehicle 45 min before injection of formalin. Pre-treatment with capsazepine suppressed the antinociceptive effect of acetaminophen. (E) The antinociceptive effect of acetaminophen (AcAP; 200 mg/kg) on both phases of the formalin test was inhibited by intracerebroventricular injection of capsazepine (100 nmol) 5 min before oral administration of acetaminophen. Data are presented as mean ± SEM (n = 5–8 per group). *P<0.05, **P<0.01, ***P<0.001 compared to vehicle.

We next studied the involvement of TRPV_1_ in the antinociceptive effect of acetaminophen in mice and rats. While acetaminophen inhibited both phases of the formalin test and increased the withdrawal thresholds in the tail immersion and the von Frey tests in TRPV_1_^+/+^ mice, it did not produce antinociception in TRPV_1_^−/−^ mice (Fig. 5 A, B and C). In rats, administration of acetaminophen at an oral dose of 300 mg/kg reduced both phases of the formalin test (Fig. 5 D). Pretreatment with capsazepine (10 mg/kg i.p.) prevented this antinociceptive effect of acetaminophen (Fig. 5 D). To address whether TRPV_1_ in brain is involved in the antinociceptive effect of acetaminophen, we injected capsazepine intracerebroventricularly 5 min before administration of acetaminophen at an oral dose of 200 mg/kg in the mouse formalin test. This treatment prevented the effect of acetaminophen on both the first and second phases of the formalin test (Fig. 5 E). These experiments demonstrate that the antinociceptive effect of acetaminophen is dependent on TRPV_1_ and indicate that TRPV_1_ in brain mediates this effect.

As shown previously in mice, acetaminophen injected subcutaneously at doses of 200 mg/kg and above reduces the PGE_2_ content in the brain and produces antinociception in the writhing test [7]. Using tandem mass spectrometry, we found that acetaminophen given intraperitoneally at a dose of 300 mg/kg reduced the content of prostanoids in the brain (PGE_2_), kidneys (PGE_2_) and blood (thromboxane B_2_), thus displaying a pharmacological profile similar to that of the COX inhibitor ibuprofen (Fig. S2 A). Furthermore, acetaminophen at this dose inhibited each phase of the formalin test to the same extent in TRPV_1_^−/−^ mice and their wild-type littermates (Fig. S2 B). Thus, a reduction of prostaglandins in brain and peripheral tissues could contribute to the TRPV_1_-independent effect of high doses of acetaminophen on the second phase of the formalin test. Although the mechanisms behind these effects remain to be determined, our study clearly shows that the antinociceptive effect of acetaminophen at an oral dose of 200 mg/kg is independent of COX, but dependent on both FAAH and TRPV_1_.

## Discussion

We have previously described a new role for FAAH in the metabolism of acetaminophen, leading to the formation of N-acylphenolamines, such as AM404, in the brain [12]. As shown *in vitro*, AM404 interferes with several important targets and mechanisms in the pain pathways, including TRPV_1_, COX and the cellular uptake and degradation of endocannabinoids. However, the relevance of this novel metabolic pathway and its downstream target(s) for the antinociceptive effect of acetaminophen remained an open question.

In the present study, we provide evidence that TRPV_1_ mediates the antinociceptive effect of acetaminophen in the formalin test in the mouse and rat as well as the tail immersion and the von Frey tests in the mouse. Furthermore, the lack of antinociception in FAAH^−/−^ mice underscores the critical role of FAAH and the potential involvement of N-acylphenolamines, such as AM404, in the antinociceptive effect of acetaminophen in rodents [6], [12]. This action of acetaminophen is independent of COX, because acetaminophen did not affect the level of prostaglandins in the brain at the dose used, and ibuprofen could not mimic the antinociceptive effect of acetaminophen in the various tests of non-inflammatory pain, although it substantially reduced the content of PGE_2_ in the brain and peripheral tissues.

Our previous observation that AM404 is produced mainly in the brain after administration of acetaminophen *in vivo* [12] prompted us to study its effect following intracerebroventricular injection in the mouse formalin test. These studies showed that AM404 produces antinociception via activation of TRPV_1_ locally in the brain. Importantly, inhibition of TRPV_1_ by an intracerebroventricular injection of capsazepine abolished the antinociceptive effect of orally administered acetaminophen, supporting the view that acetaminophen has a central site of action [10], [27]. TRPV_1_ is expressed in several brain areas of importance for nociceptive signaling, including the periaqueductal gray [28]–[30]. Interestingly, there is extensive evidence in the literature that the analgesic effect of acetaminophen in both animals and man is dependent on bulbospinal serotonergic pathways [8], [31]–[35]. Injection of capsaicin into the ventrolateral periaqueductal gray releases glutamate in the rostral ventromedial medulla, which in turn activates inhibitory bulbospinal pathways and produces antinociception [29], [36]. Our finding that acetaminophen acts via TRPV_1_ at the supraspinal level is compatible with such a mechanism, although future studies are needed to define the exact site(s) of action in the brain for this TRPV_1_-mediated effect.

Besides the brain, the spinal cord and dorsal root ganglia also express FAAH [12], [37], [38]. These tissues can catalyse the biosynthesis of AM404 *in vitro*, as shown in tissue homogenates incubated with *p*-aminophenol [12]. However, the *in vitro* formation of AM404 was much smaller in the spinal cord and dorsal root ganglia than in the brain under identical experimental conditions [12]. Furthermore, only trace amounts of AM404 could be detected in the rat spinal cord after administration of acetaminophen *in vivo* [12]. Thus, although AM404 injected intrathecally can produce antinociception in the mouse formalin test [15], it seems unlikely that AM404 via activation of TRPV_1_ in the spinal cord or dorsal root ganglia contributes to the antinociceptive effect of acetaminophen.

Recent work has shown that some antinociceptive effects of acetaminophen are lost in CB_1_^−/−^ mice and inhibited by CB_1_ receptor antagonists in rats [6], [39]. This is intriguing because, acetaminophen does not interact with the endocannabinoid system *in vitro* and fails to qualify as a cannabimimetic compound in the classical tetrad test after oral administration in rats [6], [40]. Furthermore, AM404 is a poor ligand at the CB_1_ receptor [21], [41]. In the present study, we found no effect of acetaminophen on global levels of endocannabinoids in mouse and rat brain. It is therefore unlikely that acetaminophen or any of its metabolites, including AM404, produces antinociception via inhibition of endocannabinoid uptake and degradation. In this context, it is noteworthy that TRPV_1_-mediated nocifensive responses triggered by capsaicin disappear in CB_1_^−/−^ mice or in animals subjected to pharmacological inhibition of the CB_1_ receptor [42], [43]. This indicates a functional interaction between TRPV_1_ and the CB_1_ receptor that may explain why the antinociceptive effects of acetaminophen were suppressed following genetic or pharmacological inactivation of the CB_1_ receptor [6], [39]. Clearly, more studies are needed to understand the complex interplay between TRPV_1_ and the CB_1_ receptor in the nervous system.

As early as 1972, Flower and Vane proposed that acetaminophen exerts its therapeutic effects by inhibiting prostaglandin formation in the central nervous system [44]. We found that acetaminophen at an oral dose of 200 mg/kg, which produced robust antinociceptive effects, had no effect on the content of PGE_2_ in brain. However, when administered at an intraperitoneal dose of 300 mg/kg, it was almost as effective as ibuprofen to reduce PGE_2_ contents in the brain. This dose of acetaminophen also reduced the levels of prostanoids in peripheral tissues, which does not support the view that acetaminophen inhibits COX selectively in the brain. Our studies on TRPV_1_^−/−^ mice also revealed a TRPV_1_-independent effect of this dose of acetaminophen in the formalin test. Thus, multiple dose-dependent effects may explain why there is no consensus regarding the mechanism of action of acetaminophen.

**Figure 6:**
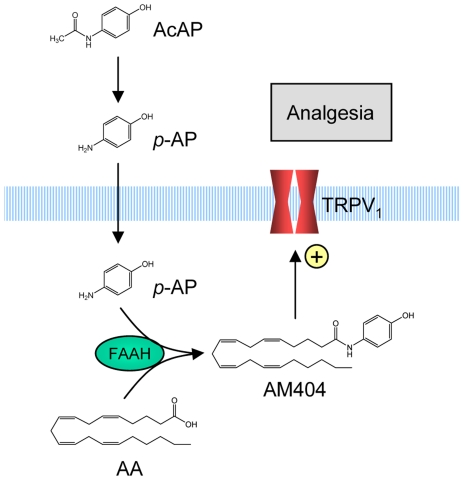
Mechanism behind the TRPV_1_-mediated antinociceptive effect of acetaminophen. Acetaminophen is metabolized to *p*-aminophenol (*p*-AP) mainly in the liver. *p*-Aminophenol is subsequently conjugated with arachidonic acid (AA) in FAAH-containing cells in the nervous system, including neurons expressing TRPV_1_, leading to the formation of the TRPV_1_ activator AM404. As suggested for capsaicin, AM404 may activate TRPV_1_ from inside of the cell [53], [54]. In contrast to AM404, acetaminophen and *p*-aminophenol do not directly interact with TRPV_1_ [12]. Activation of TRPV_1_ could produce antinociception by stimulation of bulbospinal descending inhibitory pathways in the periaqueductal gray [28].

The expression pattern in adult animals and the close evolutionary development of TRPV_1_ and FAAH implicate a functional relationship between these proteins in the central nervous system [28], [37], [45], [46]. This together with our finding that acetaminophen via its metabolite *p*-aminophenol is conjugated to AM404 in the brain [12] indicate that AM404 may be formed in TRPV_1_-containing neurons or adjacent cells in the central nervous system following administration of acetaminophen (Fig. 6). The involvement of both FAAH and TRPV_1_ in the pharmacological action of acetaminophen and the coexpression of these proteins in the brain provide a strategy for targeted delivery of TRPV_1_ activators to the nociceptive system. This may circumvent some of the feared pulmonary and cardiovascular adverse effects of systemically administered TRPV_1_ activators [47], [48]. Understanding the substrate specificity and the kinetics of fatty acid conjugation may help to develop novel analgesics that are more effective and less toxic than acetaminophen. Our study also raises the possibility that other high dose analgesic and/or anti-inflammatory agents may undergo a similar FAAH-mediated bioactivation, contributing to their pharmacological effects.

In conclusion, we provide evidence that TRPV_1_ in brain mediates the antinociceptive effect of acetaminophen and propose a strategy for developing TRPV_1_ active oral analgesics based on the coexpression of TRPV_1_ and FAAH in the central nervous system.

## Materials and Methods

### Ethics statement

All animal procedures were approved by the Regional ethics committee for animal experiments for the region Auvergne in France (CEMEA Auvergne; nr CE 0908 and CE 1010) and Malmö/Lund animal ethics committee (nr: M 26-07).

### Animals and administration of drugs

Adult C57BL/6 mice (20–30 g) of either sex were purchased from Taconic (Denmark) or Charles River Laboratories (France). Sprague-Dawley rats (175–250 g) were obtained from Charles River Laboratories (France and Germany). Only male animals were used in the nociceptive tests. TRPV_1_ and FAAH knockout mice were originally generated by David Julius [24] and Benjamin Cravatt [49]. Animals were housed under standard conditions (21–22°C; 12/12 h light/dark cycle) with food and water *ad libitum*. Acetaminophen (100, 200 or 300 mg/kg) and ibuprofen (100 mg/kg) were administered by oral or intraperitoneal injections in volumes of 10 ml/kg. The TRPV_1_ antagonist capsazepine (10 mg/kg) was administered intraperitoneally in a volume of 1 ml/kg. Drugs for intracerebroventricular (left lateral ventricle) and intraplantar (hindpaw) administration to mice were injected in volumes of 1 and 25 µl, respectively. In the biochemical studies, the animals were anesthetized by CO_2_ or isoflurane inhalation and decapitated 20 min after injection of acetaminophen or ibuprofen. Thereafter the brain, kidneys and blood were collected, snap frozen in liquid nitrogen and kept on dry ice until stored at −70°C. Blood was collected in test tubes containing 60 µl buffered citrate.

### *In vivo* tests

For assessment of locomotor activity, mice were placed in actimetry boxes (Actisystem, Apelex, Passy, France) and spontaneous motor activity was assessed by determining the number of crossings of light beams during 15 min. The test was performed 20 min after acetaminophen or vehicle (NaCl 0.9%) administration. In the formalin test, mice and rats were first acclimatized for 20 min in the test chamber. For assessment of antinociceptive effects of acetaminophen or ibuprofen in the formalin test, drugs or vehicle were administered systemically 20 min and 40 min prior to an intraplantar injection of a 2.5% formalin solution (25 µl and 50 µl) into a hindpaw in mice and rats, respectively. In the rat formalin test, acetaminophen-induced antinociception was also assessed in animals given capsazepine or its vehicle intraperitoneally 5 min prior to acetaminophen. Spontaneous biting and licking of the injected paw were monitored 0–5 min (phase 1) and 15–40 min (phase 2) in mice and 0–5 min (phase 1) and 20–40 min (phase 2) in rats after formalin injection to assess effects on both phases of the nociceptive response. In the tail immersion test, tails of mice were submerged in a water bath at 46°C until withdrawal was observed (cut-off 15 s). Four baseline latencies were measured and averaged before drug administration. Withdrawal latencies were measured 20 min after drug or vehicle administration. Calibrated von Frey filaments (0.0045–5.4950 g) was used to achieve light noxious mechanical stimulation in mice [50]. Tests were commenced after one hour of habituation. The filaments, tested in order of increasing stiffness, were applied five times perpendicular to the plantar surface of the hindpaw and pressed until bending. The first filament that evoked at least three consecutive responses was assigned as the threshold (cut-off 2 g). Drugs or vehicle was given 20 min before the start of the test. In the tail immersion and the von Frey tests, maximal possible effect (MPE) was calculated to facilitate inter-group comparisons, using a pre-determined cut-off value as follows: [(post-treatment score – pre-treatment score)/(cut-off value – pre-treatment score)]. To address whether the antinociceptive effect of acetaminophen was centrally mediated, the effect of intracerebroventricular injection of AM404 in the formalin test was investigated by injecting this compound either 5 min before or 10 min after the injection of formalin in mice. Spontaneous biting and licking of the paw was then monitored 0–5 min (phase 1) and 15–30 min (phase 2) after the formalin injection. Intracerebroventricular injections of capsazepine were also performed in mice either simultaneously with or 5 min before the administrations of AM404 (i.c.v.) and acetaminophen (p.o.), respectively. Finally, after intraplantar injection of AM404, the biting and licking behavior was recorded for 5 min. All behavioral experiments were performed in a quiet room and evaluated by a single investigator in a blinded manner. Each animal was exposed to only one treatment. Treatments were randomized in blocks and the experiments in each block were performed within the same time interval to avoid environmental influences.

### Quantification of prostanoids and endocannabinoids

Mouse brain and kidneys were homogenized in 1 ml Tris buffer (10 mM; pH 7.6), containing ethylenediaminetetraacetic acid (EDTA; 1 mM), ascorbic acid (0.3 mM), methylarachidonylfluorophosphonate (MAFP; 10 µM) and indomethacin (10 µM). Aliquots (200 µl) of blood and homogenates were precipitated with one ml ice-cold acetone, containing 0.1 µM [^2^H_8_]-labeled anandamide and 0.1 µM [^2^H_4_]-labeled PGE_2_ as internal standards. After centrifugation at 25200 g for 30 min (4°C), the supernatants were collected in polypropylene tubes and vacuum evaporated [12]. The extraction residues were reconstituted in 100 µl methanol and sample aliquots of 5 µl were injected into a Perkin Elmer 200 liquid chromatography system with an autosampler (Applied Biosystems, Norfolk, CT) coupled to an API 3000 tandem mass spectrometer (LC-MS-MS; Applied Biosystems/MDS-SCIEX, Toronto, Canada) [12]. A Genesis C_8_ column (20×2.1 mm; Jones, Lakewood, CO) was used for all analyses. For analysis of prostanoids, a gradient in the mobile phase was applied in 6 min starting at 25% and ending at 100% methanol. The column was then kept at 100% methanol for three min. The column was reconditioned in 25% methanol for two min. The electrospray interface was operating in the positive ion mode at 370°C and the ion spray voltage was −4000 volts. M/z 351.3/271.0 with a collision energy (CE) of −26 volts and a declustering potential (DP) of −35 volts was used for determination of PGE_2_. M/z 355.3/275.3 (CE −26 volts, DP −38 volts) and m/z 369.2/195.0 (CE −17 volts, DP −27 volts) were used for determination of [^2^H_4_]-labeled PGE_2_ and thromboxane B_2_, respectively. For analysis of endocannabinoids, a gradient in the mobile phase was applied in 6 min starting at 75% and ending at 100% methanol. The column was then reconditioned in 75% methanol for two min. The electrospray interface was operating in the positive ion mode at 370°C, and the ion spray voltage and DP were set to 5000 volts and 40 volts, respectively. M/z 348.2/62.0 (CE 35 volts), 356.4/63.0 (CE 35 volts), m/z 300.5/62 (CE 35 volts) and m/z 379.2/287.0 (CE 15 volts) were used for the determinations of anandamide, [^2^H_8_]-labeled anandamide, N-palmitoylethanolamide and 2-arachidonoylglycerol, respectively. The contents of these lipids were expressed either in mol or as normalized peak areas (nPA) and related to the protein content in the samples. The nPA was obtained by dividing the peak area for the analytes with the peak area for the internal standard in the same sample. Since 2-arachidonoylglycerol is non-enzymatically converted to 1-arachidonoylglycerol, the content of 2-arachidonoylglycerol was estimated as the sum of these lipids [51], [52]. The detection limits were calculated as the concentration corresponding to three times the standard deviation of the blanks. When levels were below detection limit, numerical values of half the detection limit were used in the calculations.

### Calculations and statistics

Data are presented as means ± standard error of the mean, and *n* indicates the number of animals used. The percent reduction of prostanoid contents was calculated from the logarithm transformed values. GraphPad Prism 5 software (GraphPad Software, San Diego, CA) was used for drawing graphs. Mann-Whitney U-test and Wilcoxon signed rank test were used for statistical analysis of paired and unpaired data. Statistical significance was accepted when P<0.05.

### Drugs

Acetaminophen, ibuprofen (Sigma-Aldrich, Lyon or Stockholm, France or Sweden) and indomethacin (Confortid®, Dumex, Copenhagen, Denmark) were dissolved in and diluted with saline or water. Capsazepine (Sigma-Aldrich) was dissolved in 10% dimethyl sulfoxide (DMSO) in saline for systemic administration or in 10% DMSO/2.5% Tween 80 in saline for intracerebroventricular administration. AM404 (Tocris Bioscience, Bristol, UK) was dissolved in 10% DMSO/2.5% Tween 80 in saline. Prostaglandin E_2_, anandamide, N-palmitoylethanolamide (Biomol, International LP, Exeter, U.K.), [^2^H_4_]-labeled PGE_2_, [^2^H_8_]-labeled anandamide, 2-arachidonoylglycerol, thromboxane B_2_ and MAFP (Cayman Chemical, Ann Arbor, M.I.) were all dissolved in and diluted with ethanol.

## Supporting Information

Figure S1 Effect of acetaminophen on endocannabinoid contents in rat brain. The contents of anandamide (AEA) and 2-arachidonoylglycerol (2-AG) did not differ between animals exposed to acetaminophen (300 mg/kg p.o.) or vehicle for 15 min. Y-axis shows values as normalized peak area (nPA), obtained by dividing the peak area for the analytes with the peak area for the internal standard (0.1 µM d8-anandamide) in the sample. Data are given as mean ± SEM (n = 6).
(2.09 MB TIF)

Figure S2 A high intraperitoneal dose of acetaminophen reduces prostanoid contents and evokes TRPV1-independent antinociceptive effects in mice. (A) Acetaminophen (300 mg/kg i.p.) and ibuprofen (Ibu; 100 mg/kg i.p.) significantly reduced the content of prostaglandin E2 (PGE2) or thromboxane B2 (TXB2) in brain, kidney and blood. Tissues were collected 20 min after injection of drug or vehicle. The reduction is presented as a percentage of the vehicle for each group. Data are given as mean ± SEM (n = 6). **P<0.01 compared to vehicle treated animals. (B) The effect of acetaminophen (300 mg/kg i.p.; AcAP) in the formalin test was intact in TRPV1^−/−^ mice. Acetaminophen was administered 20 min before intraplantar injection of formalin. Data are given as mean ± SEM (n = 6). **P<0.01 compared to vehicle.
(4.32 MB TIF)

Table S1 Contents of endocannabinoids (pmol/mg protein) in the mouse brain 20 min after injection (i.p.) of vehicle or acetaminophen at a dose of 300 mg/kg. Data are given as mean ± SEM (n = 6).
(0.03 MB DOC)
