# Supplementary figures and images for: TRPV1 in Brain Is Involved in Acetaminophen-Induced Antinociception
Source: PLoS One. 2010 Sep 17;5(9):e12748. doi: 10.1371/journal.pone.0012748 (PMC2941447; doi:10.1371/journal.pone.0012748)

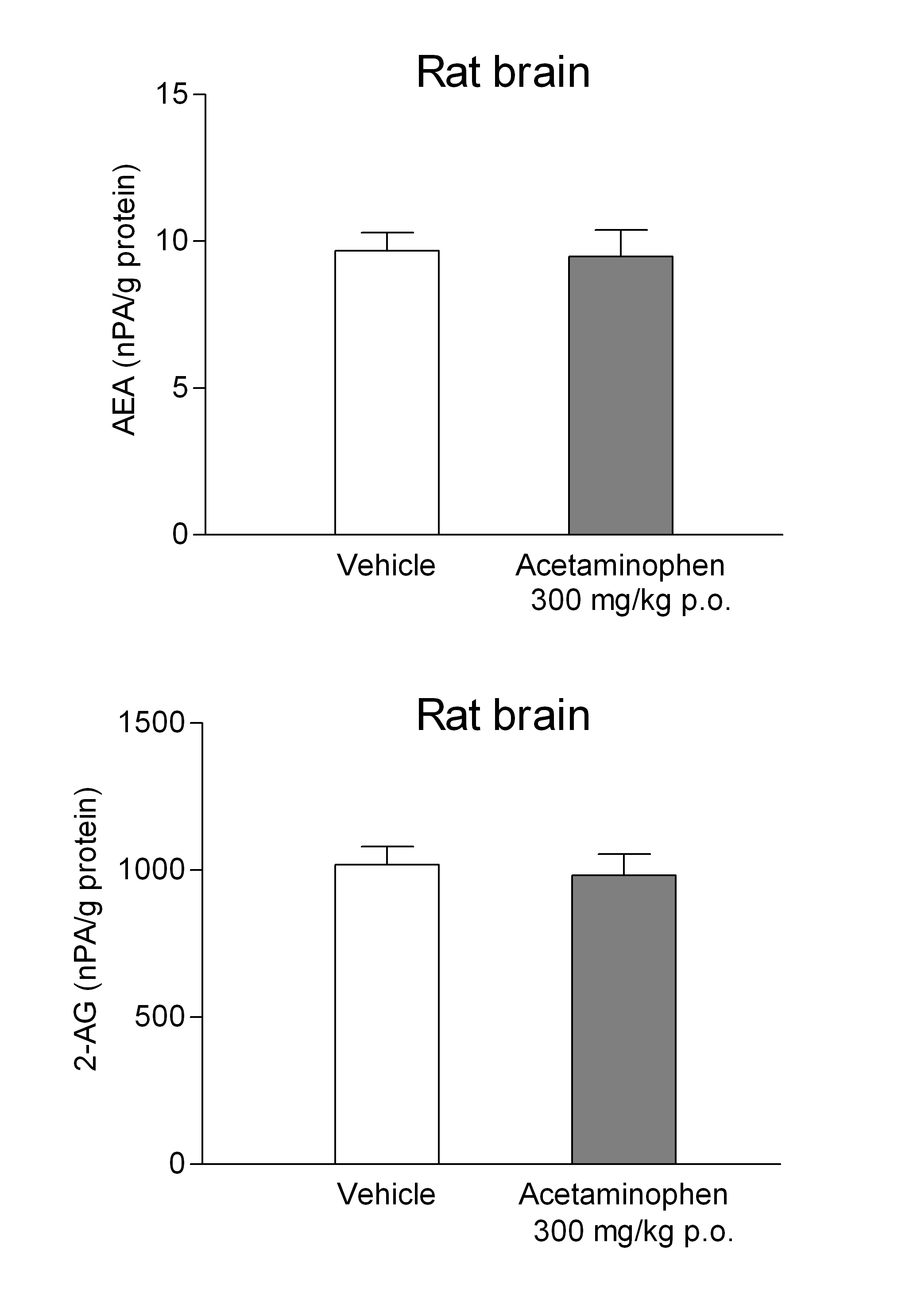

Supplement: Figure S1 — Effect of acetaminophen on endocannabinoid contents in rat brain. The contents of anandamide (AEA) and 2-arachidonoylglycerol (2-AG) did not differ between animals exposed to acetaminophen (300 mg/kg p.o.) or vehicle for 15 min. Y-axis shows values as normalized peak area (nPA), obtained by dividing the peak area for the analytes with the peak area for the internal standard (0.1 µM d8-anandamide) in the sample. Data are given as mean ± SEM (n = 6). (2.09 MB TIF) [file pone.0012748.s001.tif]

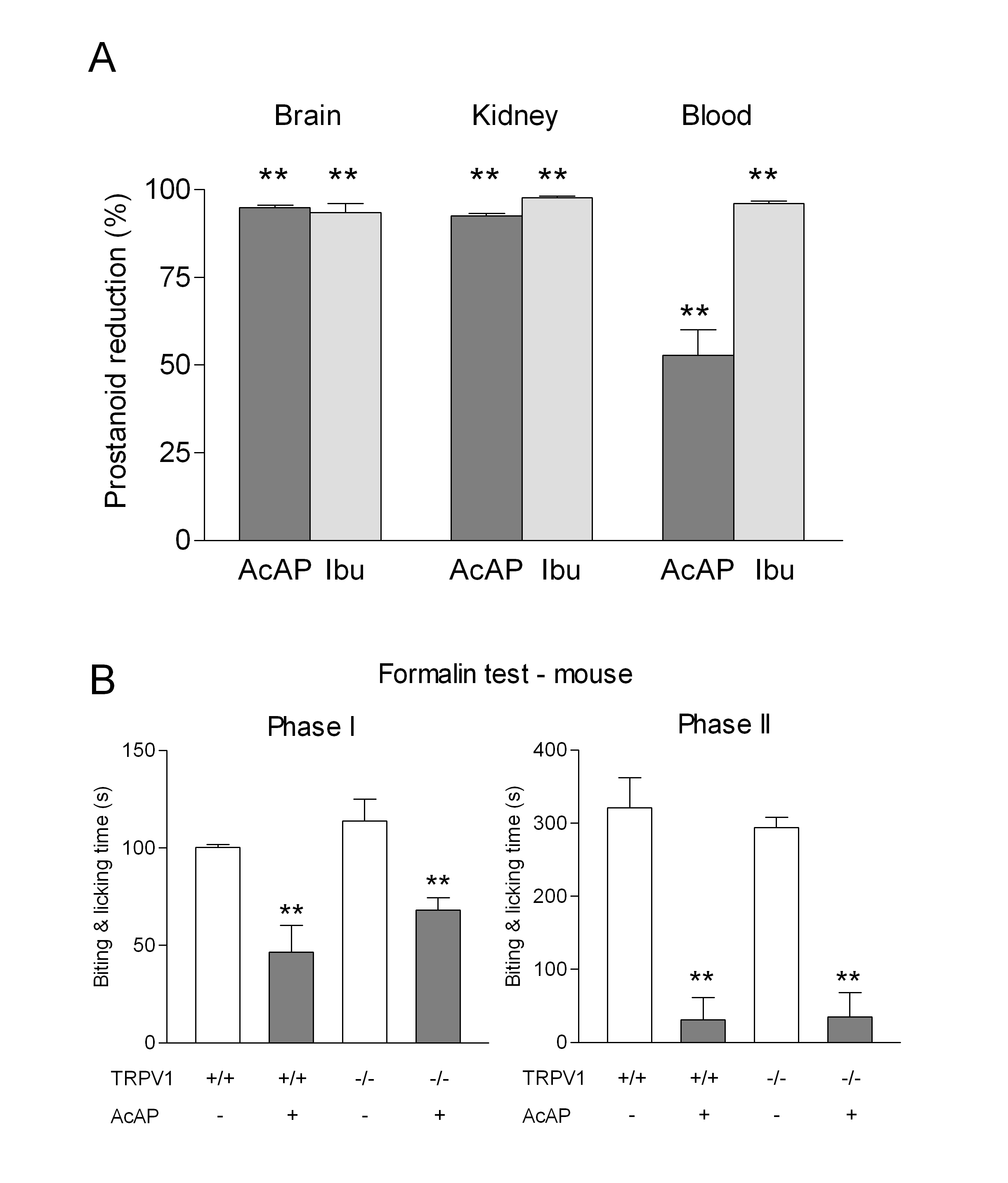

Supplement: Figure S2 — A high intraperitoneal dose of acetaminophen reduces prostanoid contents and evokes TRPV1-independent antinociceptive effects in mice. (A) Acetaminophen (300 mg/kg i.p.) and ibuprofen (Ibu; 100 mg/kg i.p.) significantly reduced the content of prostaglandin E2 (PGE2) or thromboxane B2 (TXB2) in brain, kidney and blood. Tissues were collected 20 min after injection of drug or vehicle. The reduction is presented as a percentage of the vehicle for each group. Data are given as mean ± SEM (n = 6). **P<0.01 compared to vehicle treated animals. (B) The effect of acetaminophen (300 mg/kg i.p.; AcAP) in the formalin test was intact in TRPV1−/− mice. Acetaminophen was administered 20 min before intraplantar injection of formalin. Data are given as mean ± SEM (n = 6). **P<0.01 compared to vehicle. (4.32 MB TIF) [file pone.0012748.s002.tif]
