# Supplementary material for: TRPV1 in Brain Is Involved in Acetaminophen-Induced Antinociception
Source: PLoS One. 2010 Sep 17;5(9):e12748. doi: 10.1371/journal.pone.0012748 (PMC2941447; doi:10.1371/journal.pone.0012748)
Supplement: Table S1 — Contents of endocannabinoids (pmol/mg protein) in the mouse brain 20 min after injection (i.p.) of vehicle or acetaminophen at a dose of 300 mg/kg. Data are given as mean ± SEM (n = 6). (0.03 MB DOC) [file pone.0012748.s003.doc]

| Endocannabinoid | Vehicle | Acetaminophen |
| --- | --- | --- |
| Anandamide | 0.4 ± 0.04 | 0.4 ± 0.07 |
| Palmitoylethanolamide | 6.0 ± 0.2 | 6.2 ± 0.3 |
| 2-Arachidonoylglycerol | 1300 ± 98 | 1100 ± 64 |
